# Supplementary material for: Perceptions of Body Condition, Diet and Exercise by Sports Dog Owners and Pet Dog Owners
Source: Animals (Basel). 2021 Jun 11;11(6):1752. doi: 10.3390/ani11061752 (PMC8230856; doi:10.3390/ani11061752)
Supplement: Supplementary file 1 [file animals-11-01752-s001.zip › animals-1213383 supplementary for conversion.pdf]

Supplementary Data and Discussion

Results

Owners

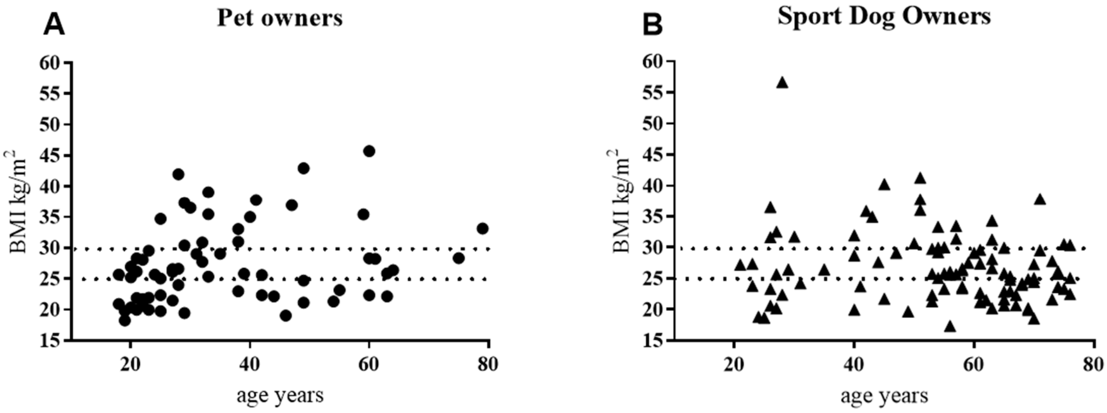

Figure S1. Individual values for Body Mass Index by age in the pet owner group (A) and the sport owner group (B). Each point represents an individual. Horizontal lines indicate overweight (25–29.99kg/m^2) and obese (>30kg/m^2) BMI classifications.

The owner BMI (calculated from owner reported height and weight) was not different in the pet dog group (27.2±6.5 kg/m^2) and the sport dog group (26.3±6.5 kg/m^2). See Figure 1 for BMI by age for each group. The percentage of participants in the healthy BMI category was 39% for the pet owners and 44% for the Sport dog owners. The number of participants classified as obese was 26% (pet dog owners) and 22% (sport dog owners). The median income for pet owners was $50,000-70,000 and the median education was University (undergraduate degree). For the sport dog owners the median income was $90,001-120,000 and the median education was University (undergraduate degree). Only 7 sport dog owners and 16 pet dog owners reported having children in the household.

Dogs

See Table S1 for the owner reported breeds by group.

Table S1. Dog Breeds by group.

Dog Breed	Number Pet Dogs	Number Sports Dogs
American Bulldog	1	1
American Pitbull	1	
American Staffordshire Terrier	1	
American Eskimo Dog		1
Australian Cattle Dog		2
Australian Shepherd	2	10
Belgian Malinois		2
Border Collie		10
Boston Terrier	3	2
Boxer	1	1
Brittany Spaniel	2	
Catahoula Leopard Dog		1
Chihuahua	1	
Dachshund	2	
Deutsch Drahthaar	1	
Doberman Pinscher		3

English Springer Spaniel	1	2
German Shepherd	2	3
German short hair pointer	1	
Great Dane	1	
Golden Retriever	2	5
Labrador Retriever	1	2
Maltese	2	
Mini American Shepherd		1
Miniature Pinscher	1	
Miniature Poodle		1
Miniature Schnauzer	1	1
mixed breed	35	11
Nova Scotia Duck Tolling Retriever		2
Papillion		1
Parson Russell Terrier		2
Pembroke Welsh Corgi		1
Portuguese Water Dog		23
Pyrenean Shepherd		1
Rottweiler	1	
Shetland Sheep Dog		8
Shih Tzu	1	
Standard poodle	1	
Standard Rat Terrier		1
Standard Schnauzer		1
Swedish Valhund		2
Toy Poodle		1
Yorkshire Terrier	1	

See Table S2 for reported brands by group)

Table S2. Pet food brands reported by group.

	Pet Dogs	Sports Dogs
4Health		2
All Provide		2
Blue Buffalo	1	
Blue Ridge Beef		3
Blue Wilderness		1
Bravo		2
Canidae	1	1
Chicken Soup for the Soul	1	
Diamond Naturals	2	1
Earthborn Holistic		2
Farmina		2
Fresh Pet		1
Fromm	1	7
Great Choice	1	
Holistic		1
Honest Kitchen	1	2
Iams	1	2
Instinct	2	2
Merrick	1	2
Natural Balance		1
Natures Logic	1	1
Natures Recipe	1	
Natures Variety		2
Northwest Naturals		1
Now	1	

Rachel Ray Nutrish	2	1
Nutro		1
Nutroma X	1	
Orijen	1	2
Primal		5
Royal Canin	7	
Science diet	1	
Sojos mixed with dry		1
Sport dog food		3
Sportman's pride Maintenance		1
Stella and Chewy's		2
Taste of the Wild	1	3
Tuckers		1
Ulta		1
Venture Alaskan Pollock		1
Victor	1	4
Wellness Core		5
Zignature	1	

Questionnaires

The questions by category are presented in table S1 and S2. Table S1 are the results from the feeding portion of the questionnaire and table S2 is from the exercise portion of the questionnaire.

Table S3. Feeding Beliefs and Intentions.

	**Average score Sport dog group N=101**	**Average score Pet dog group N=70**	**p**	**Effect size Cohens D**	**Cronbach's alpha**
**Feeding barriers external owner**<br>My dog is overfed because others feed the dog.<br>My dog is overfed because he/she is always wants food.<br>My dog isn't given the appropriate type of food because others feed the dog.<br>I feed my dog inappropriate types of food because he/she likes that kind of food.<br>My dog isn't fed the appropriate number of times a day because others feed the dog.<br>I don't have time to feed my dog more frequent meals during the day.	1.7±0.7	2.2±1.0	0.0001	-0.57	0.71
**Feeding control**<br>Overall, how much control do you feel you have over the amount you feed your dog?<br>Overall, how much control do you feel you have over the type of food you feed your dog?<br>Overall, how much control do you feel you have over the number of times you feed your dog during the day?	6.7±0.8	6.4±0.9	0.037	0.33	0.71
**Feeding norms comply with others**	2.9±1.3	2.7±1.2	NS	0.16	0.79

I would like to feed my dog the amount that is recommended to me by other dog owners.<br>I would like to feed my dog the type of food that is recommended to me by other dog owners.<br>I would like to feed my dog as often as is recommended to me by other dog owners.					
**Feeding norms comply with vet**<br>I would like to feed my dog the amount that is recommended to me by my veterinarian.<br>I would like to feed my dog the type of food that is recommended to me by my veterinarian.<br>I would like to feed my dog as often as is recommended to me by my veterinarian.	4.1±1.4	6.0±1.0	0.0001	-1.55	0.86
**Feeding norms other**<br>My veterinarian believes that I feed my dog too much.<br>Other dog owners believe that I feed my dog too much.<br>My veterinarian believes that I don't feed my dog the appropriate type of food.<br>Other dog owners believe that I don't feed my dog the appropriate type of food.<br>Other dog owners believe that I should feed my dog more frequent meals during the day.<br>My veterinarian believes that I should feed my dog more frequent meals during the day.	2.0±0.9	2.3±0.9	0.024	-0.35	0.76

Mean ± standard deviation.

Table S4. Exercise beliefs and intentions.

	Average score Sport dog group N=101	Average score Pet dog group N=70	p	Effect size Cohens D	Cronbach's alpha
**Exercise beliefs external**<br>My dog isn't exercised for long enough because others exercise the dog.<br>My dog isn't exercised frequently enough because others exercise the dog.<br>My dog isn't given the appropriate type of exercise because others exercise the dog.	1.6±0.8	2.1±1.1	0.001	-0.51	0.85
**Exercise beliefs owner**<br>I don't exercise my dog for long enough because I don't like to.<br>I don't exercise my dog for long enough because I am physically unable to.	1.8±0.8	2.2±0.8	0.002	-0.46	0.78

I don’t exercise my dog frequently enough because I don’t like to.<br>I don’t exercise my dog as frequently as I should because I don’t have time.<br>I don’t exercise my dog as frequently as I should because he/she is badly behaved.<br>I don’t give my dog the appropriate type of exercise because I don’t like to.<br>I don’t give my dog the appropriate type of exercise because he/she doesn’t like that type.<br>I don’t give my dog the appropriate type of exercise because I don’t have access to the appropriate areas.					
**Exercise beliefs value**<br>It is important that I exercise my dog for the appropriate length of time.<br>My dog doesn’t need to be exercised.<br>It is important to me that my dog is fit<br>It is important I exercise my dog the appropriate number of times a week.<br>It is important that I give my dog the appropriate type of exercise.	6.5±0.6	6.3±0.7	0.017	0.38	0.72
**Exercise control**<br>Overall, how much control do you feel you have over the length of time you exercise your dog?<br>Overall, how much control do you feel you have over how frequently you exercise your dog?<br>Overall, how much control do you feel you have over the type of exercise you give your dog?	6.6±0.9	6.2±1.0	0.007	0.43	0.95
**Exercise intentions**<br>How likely is it, in the future that you will exercise your dog for the appropriate length of time?<br>How likely is it in the future that you will exercise your dog the appropriate number of times a week?<br>How likely is it, in the future that you will give your dog the appropriate type of exercise?	6.6±0.7	6.2±0.8	0.0001	0.56	0.89
**Exercise lack of knowledge**<br>I don’t know what the appropriate length of time my dog should be exercised.<br>I don’t know how often I should exercise my dog.<br>I don’t know what type of exercise to give my dog.	2.0±1.0	3.2±1.5	0.0001	-0.92	0.85
**Exercise norms comply with others**<br>I would like to exercise my dog for the length of time that is recommended to me by other dog owners.	3.4±1.4	3.2±1.5	NS	0.14	0.85

I would like to exercise my dog as frequently as other dog owners recommend.<br>I would like to give my dog the type of exercise that is recommended to me by other dog owners.					
**Exercise norms comply with vet**<br>I would like to exercise my dog for the length of time that is recommended to me by my veterinarian.<br>I would like to exercise my dog as frequently as my veterinarian recommends.<br>I would like to give my dog the type of exercise that is recommended to me by my veterinarian.	4.6±1.2	6.1±0.8	0.0001	-1.4	0.89
**Exercise norms other**<br>My veterinarian believes that I don't exercise my dog as frequently as I should.<br>Other dog owners believe that I don't exercise my dog as frequently as I should.<br>My veterinarian believes that I don't give my dog the appropriate type of exercise.<br>Other dog owners believe that I don't give my dog the appropriate type of exercise.<br>My veterinarian believes that I don't exercise my dog for the appropriate length of time.<br>Other dog owners believe that I don't exercise my dog for the appropriate length of time.	2.0±1.0	2.6±1.0	0.00001	-0.66	0.89

Mean ± standard deviation.

Discussion

Owners

We used owner reported height and weight to calculate Body Mass Index (BMI) and found that the average of both groups were in the overweight category. However, our participants' BMI was lower than the average BMI in the US (29 kg/m^2) (CDC NHANES 2015-2016). According to the CDC (Hales, Carroll, Fryar, & Ogden, 2020), the prevalence of obesity in the US is 40%. In our participants, less than 30% were classified as obese. Coleman et al. (Coleman et al., 2008) also reported fewer people were classified as obese in the group that walked with their dog. However, the highest rate of obesity was in the pet owners that did not walk with their dogs. This was supported by Lentino et al. (Lentino, Visek, McDonnell, & DiPietro, 2012) that also showed the highest BMI in people that owned a dog, but did not walk the dog. This suggests that pet ownership alone does not help with obesity, but walking with your dog may improve health.

Dog Body Condition

The differences among the groups of dogs was only significant with the Purina Body Condition score and the percent fat measurement. The BMI calculation did not show a difference among the groups. We saw a weak/moderate correlation between the Purina body condition score and the percent fat calculation, but poor correlations between BMI and the other body condition variables. This is in general agreement with Mawby et al.

(Mawby et al., 2004) that compared BMI, percent fat, body condition scores to DEXA body fatness measurements. Mawby et al. (Mawby et al., 2004) found a high correlation between the DEXA measurements and the Body Condition Scores and the percent fat measurements, but low/moderate correlation with BMI. They also found a high correlation between Body Condition Scores and percent fat measurements. This suggests that Body Condition Scores and percent fat calculations may be superior to BMI, when determining body condition of dogs.

Dog feeding

In the pet dog group, most of the owners reported feeding dry kibble, but only less than 1/3 of sport dog owners reported feeding dry kibble. The median brand for both groups was Purina. Out of the 44 brands written in (both groups combined), only 12 of the brands were reported by both groups. The sport group had a large percentage that wrote in feeding a raw diet, but the raw diet was not reported at all in the pet dogs. The higher prevalence of raw feeding may be a result of research suggesting that raw feeding changes the microbiome in comparison to kibble feeding (Lefebvre, Reid-Smith, Boerlin, & Weese, 2008; Sandri, Dal Monego, Conte, Sgorlon, & Stefanon, 2017; Schmidt et al., 2018) and anecdotal evidence that it improves dog health.

Coleman, K. J., Rosenberg, D. E., Conway, T. L., Sallis, J. F., Saelens, B. E., Frank, L. D., & Cain, K. (2008). Physical activity, weight status, and neighborhood characteristics of dog walkers. *Preventive Medicine, 47*(3), 309-312. doi:10.1016/j.ypmed.2008.05.007

Hales, C. M., Carroll, M. D., Fryar, C. D., & Ogden, C. L. (2020). Prevalence of Obesity and Severe Obesity Among Adults: United States, 2017-2018. *NCHS Data Brief*(360), 1-8.

Lefebvre, S. L., Reid-Smith, R., Boerlin, P., & Weese, J. S. (2008). Evaluation of the risks of shedding Salmonellae and other potential pathogens by therapy dogs fed raw diets in Ontario and Alberta. *Zoonoses and Public Health, 55*(8-10), 470-480. doi:10.1111/j.1863-2378.2008.01145.x

Lentino, C., Visek, A. J., McDonnell, K., & DiPietro, L. (2012). Dog Walking Is Associated With a Favorable Risk Profile Independent of a Moderate to High Volume of Physical Activity. *Journal of Physical Activity & Health, 9*(3), 414-420. doi:DOI 10.1123/jpah.9.3.414

Mawby, D. I., Bartges, J. W., d'Avignon, A., Laflamme, D. P., Moyers, T. D., & Cottrell, T. (2004). Comparison of various methods for estimating body fat in dogs. *Journal of the American Animal Hospital Association, 40*(2), 109-114. doi:Doi 10.5326/0400109

Sandri, M., Dal Monego, S., Conte, G., Sgorlon, S., & Stefanon, B. (2017). Raw meat based diet influences faecal microbiome and end products of fermentation in healthy dogs. *Bmc Veterinary Research, 13*. doi:ARTN 65

10.1186/s12917-017-0981-z

Schmidt, M., Unterer, S., Suchodolski, J. S., Honneffer, J. B., Guard, B. C., Lidbury, J. A., . . . Kolle, P. (2018). The fecal microbiome and metabolome differs between dogs fed Bones and Raw Food (BARF) diets and dogs fed commercial diets. *PLoS One, 13*(8). doi:ARTN e0201279

10.1371/journal.pone.0201279
